# Aging in magma rheology

**DOI:** 10.1038/s41598-022-14327-2

**Published:** 2022-06-15

**Authors:** Aika K. Kurokawa, Takahiro Miwa, Hidemi Ishibashi

**Affiliations:** 1 National Research Institute for Earth Science and Disaster Resilience, 3-1 Tennodai, Tsukuba, Ibaraki 3050006 Japan; 2 Department of Geosciences, Faculty of Science, Shizuoka University, 836 Ohya, Suruga-ward, Shizuoka, 4228017 Japan

**Keywords:** Volcanology, Geophysics

## Abstract

Aging, change in property depending on the elapsed time from preparation, is known to affect the rheological behavior of various materials. Therefore, whether magma ages must be examined to characterize potentially widespread volcanic phenomena related to the transition from rest to flow. To achieve this, we performed rheological measurements and microstructural analyses on basaltic andesite lava from the 1986 Izu-Oshima eruption. The rheology shows an initial overshoot of shear stress during start-up flow that correlates with the duration and the shear rate of a pre-rest time. This indicates that the yield stress of magma and lava increases with aging. The microstructure shows that original aggregates of crystals, which may grow during crystallization, coalesce during the pre-rest period to form clusters without changing the crystal volume fraction. We conclude that the clusters are broken by shear in the start-up flow, which induces the stress overshoot. Thus, aging in magma rheology will impact the understanding of dynamic flow.

## Introduction

Magma rheology is a key to understand widespread volcanic phenomena related to the flow of magma and lava; therefore, there have been many studies on it, as reviewed by^1,2^. The rheological behavior of magma is controlled not only by temperature, pressure, and chemical composition, but also by the presence of crystals and bubbles^3–7^. The effect of crystals is especially influential, and the volume of crystals increases with cooling and/or degassing^8,9^.

The crystal volume fraction is a critical parameter to describe the rheology of magma composed of crystals and melt. At low crystal volume fraction, magma behaves as a Newtonian fluid, whereas with an increase in the crystal volume fraction, the rheology of magma becomes complicated and non-Newtonian^5,10,11^. To date, major non-Newtonian behaviors have been examined at the steady state to extract one-to-one relationships, such as viscosity vs. crystal volume fraction^12^ and yield stress vs. crystal volume fraction^13^, because relational equations are required for simulations of magma and lava flow^4,14^. Equations to predict the effect of crystal shape on rheology are also proposed and widely applied^1,15,16^.

In contradiction to the formulated characteristics at the steady state, non-Newtonian rheology at non-steady state such as the start-up of flow is more complex. Previous studies have reported thixotropy^11,17–21^, which is the continuous decrease of viscosity with time when flow is applied to a sample that has been previously at rest and the subsequent recovery of viscosity in time when the flow is discontinued^22,23^. Although one explanation for the behavior is temporal alignment change of plagioclase crystals with shear^20,24,25^, the relationship between the rheology and the microstructure is less well understood.

As time-dependence, aging, or change in property depending on the elapsed time since a sample was prepared, has been under intense study in the fields of nonlinear physics and soft matter, because it has strong effects on rheology^26–29^. Many soft solids, such as polydomain defect textures in ordered mesophases of copolymers or surfactants may show rheological aging through coarsening dynamics, or through glassy rearrangement of domains, or both^26^. In the same manner, melt-crystal textures of magma can vary with time, generating transition from rest to flow and the associated rheological evolution such as changes in thixotropy. However, there is no study that has examined the appearance of aging in magma until now.

We performed experiments to shed some light on aging in magma rheology using lava samples from the 1986 Izu-Oshima eruption. We report rheological changes as functions of time and shear rate of pre-rest, during which the sample rests before the measurement. The results are combined with the microstructures of quenched samples under different conditions for pre-rest to discuss the relationship between the rheology and the microstructure in terms of aging. Finally, we discuss how the aging in magma rheology may practically affect the dynamic flow at a volcano.

## Materials and methods

### Sample preparation

The starting material for this study was a rock sample from lava produced by the 1986 eruption at Izu-Oshima, an isolated volcanic island, 110 km SSW of Tokyo, Japan. The eruptive sequence comprised three eruptions at summit crater A, fissures in the caldera floor (crater B), and fissures in the flank of the outer rim (crater C) in chronological order^30^. We used lava from fissure crater B (LB) because the aphyric basaltic andesite fully melts in a relatively short period of time and has a sufficiently low viscosity that is measurable within the mechanical constraints of our experimental system. The chemical data shown in Table S1 of the Supplementary Material is consistent with those of the other LB samples collected within one year after the eruption^31,32^. The rock sample was crushed and sieved to a particle size less than 4 mm for the experiment.

### Experimental apparatus

The experimental system used in this study is an improved version of that described by^33^. The furnace was changed to a high-temperature muffle furnace (MSFS-1218, Yamada Denki Co., Ltd.) that can increase the temperature up to 1400°C. The LB sample was placed in an alumina crucible with 17% porosity. Porous alumina was selected to prevent fracture during water-quenching and to maximize the quenching rate. A concentric cylindrical viscometer (HBDV-II+Pro, Brookfield Engineering Laboratories, Inc.) was connected to an alumina rod introduced into the LB sample from a hole on the top of the furnace to measure the torque. To calculate the shear stress and the shear rate, the equations described in^33^ were used with the sample height (15 mm), the radius of the rod (2.5 mm), and the radius of container (9.5 mm). Note that the reaction between the alumina parts and the LB sample is limited to the interface and does not affect either the overall microstructure of the sample or the calculation of shear rate. Further details about the calculations are described in Text S1 of the Supplementary Material.

### Experimental protocol

Experiments were performed in an atmospheric environment at 1 atm with the following steps. (1) The furnace was heated to 1300°C and the temperature was maintained for 6 h to achieve complete melting of the starting material. (2) A rod was inserted into the sample 3 h after heating was started in (1) and weak shear at 1.13 s^−1^ was imposed for separating gas between fragments of the sample gravitationally and tracking the rheological change. (3) The temperature was lowered to the experimental temperature of 1180°C and shearing at the rate imposed in (2) was continued until the sample has the equilibrium crystal content at the PT conditions. The temporal variation in the shear stress during the process is indicated in Fig. S1 of the Supplementary Material. The temperature of 1180°C, which is between the solidus and the liquidus, was selected to observe the effect of microstructural changes in crystals on the rheology. It is 80°C higher than the eruption temperature of LB, 1100°C estimated by^31^. (4) Preshearing was applied at 4.50 s^−1^ for 2 min. The shear rate was higher than that of all tests to initialize and homogenize the sample. (5) Pre-rest was set for $t_{pr}$ to relax the sample before measurement, and allow recovery of the microstructure, or aging if it shows. During the pre-rest, zero or weak shear ($\dot{\gamma }_{pr} \le$ 0.45 s^−1^) was imposed to examine if aging occurs with shear. (6) A shear-rate controlled test (SRC) was performed at a shear rate ($\dot{\gamma }_{src}$) within the range of 0.68−2.48 s^−1^, which is equivalent to that of actual lava flows^34,35^. (7) Processes (4)–(6) were repeated with changing $t_{pr}$, $\dot{\gamma }_{pr}$, and $\dot{\gamma }_{src}$ to examine the rheological variations in magma induced by aging. After the series of SRCs, the rod was gently pulled out from the sample, and the sample was immediately quenched in water for observation using scanning electron microscopy (SEM; JSM-IT500, JEOL) and for micro-computed tomography (micro-CT; SkyScan 1272, Bruker).

## Results and discussion

### Rheology

#### Effect of pre-rest time

**Figure 1:**
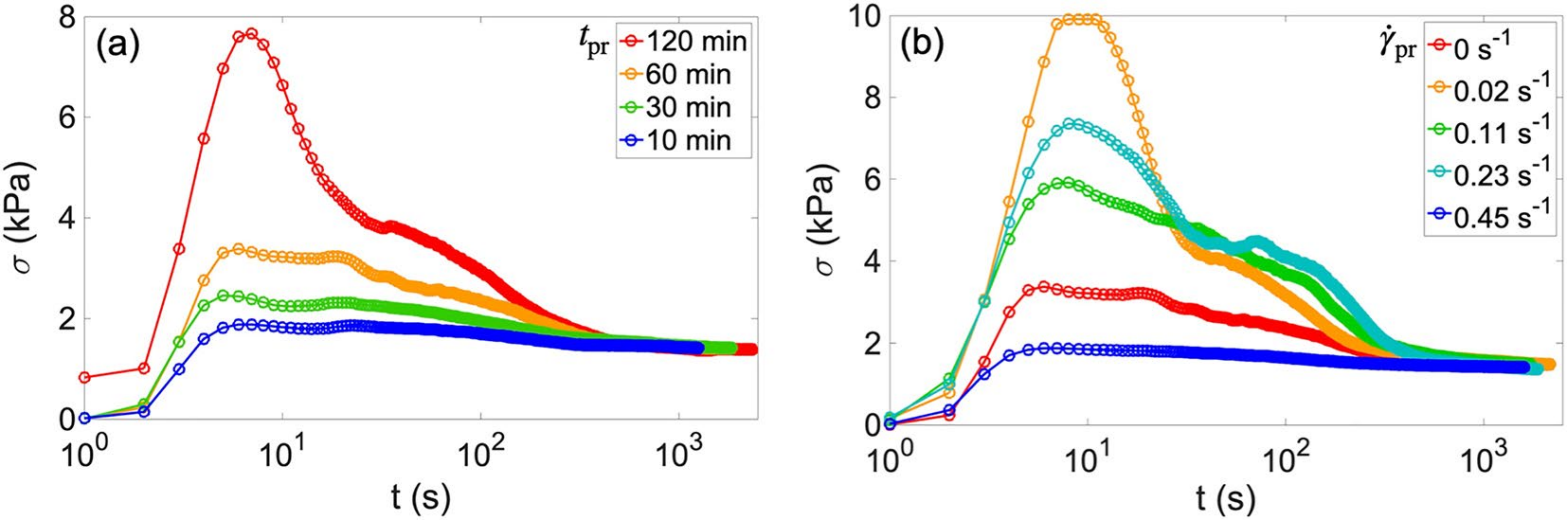
(**a**) Results of SRC tests at $\dot{\gamma }_{src}$ of 1.13 s^−1^ after various pre-rest times $t_{pr}$, as shown in the legend. The sample was not sheared during the pre-rest times ($\dot{\gamma }_{pr}$ = 0 s^−1^). (**b**) Results of SRC tests at $\dot{\gamma }_{src}$ of 1.13 s^−1^ after t$_{pr}$ = 60 min at various $\dot{\gamma }_{pr}$, as shown in the legend.

Figure 1a presents the effect of the pre-rest time ($t_{pr}$) on the subsequent SRC test. The shear stress relaxes smoothly towards a constant value after the shortest $t_{pr}$ of 10 min. For longer $t_{pr}$, the stress increases toward a peak in the start-up flow and falls to be a constant through the overshoot. The stress overshoot becomes pronounced, or the peak stress increases with $t_{pr}$. The rheological variation with $t_{pr}$ is evidence that magma shows aging. On the other hand, the stress eventually matches after the long amount of time/strain ($\sim$ 400 s in Fig. 1a), regardless of $t_{pr}$. The agreement in the final stress indicates the one-to-one relation between the shear stress and the shear rate at steady state that has been reported in many other magmatic systems to date^7,33^. Therefore, magma of which steady state seems simple, can show complex rheology at non-steady state due to aging.

#### Effect of shear rate during pre-rest

The occurrence of aging with shear is examined by changing $\dot{\gamma }_{pr}$ at the pre-rest time of 60 min, which is adequate to observe the aging effect, as illustrated in Fig. 1a. Figure 1b indicates that the stress overshoot becomes more predominant with low $\dot{\gamma }_{pr}$ than that without shear, while the shear stress remains constant without the experience of the overshoot at the highest $\dot{\gamma }_{pr}$ of 0.45 s^−1^. This indicates that aging occurs at $\dot{\gamma }_{pr}$ below 0.45 s^−1^ in this case, although the threshold may vary depending on the shear rate of the SRC test, and the crystal variations and shapes. There may be competition between aging and shear-rejuvenation (viscosity decrease in time under shear^28^) during the pre-rest process as it in flows of soft matters^28,29^. Aging is promoted by weak shear, while rejuvenation wins at high shear rate and diminishes the stress overshoot. Therefore, $\dot{\gamma }_{pr}$ is also a key parameter to induce the aging effect as well as $t_{pr}$.

#### Effect of shear rate in SRC test

**Figure 2:**
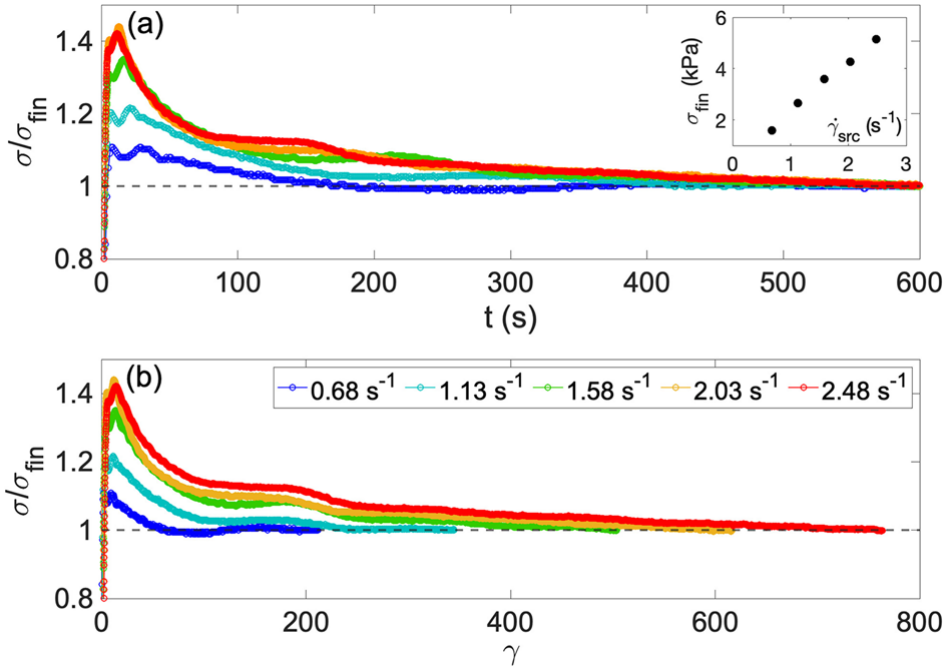
Results of SRC tests at various shear rates after pre-rest with t$_{pr}$ of 60 min and $\dot{\gamma }_{pr}$ of 0 s^−1^. $\dot{\gamma }_{src}$ are indicated by the colors in the legend of (**b**). $\sigma _{fin}$ is the average value of the last 10 s. (**a**) Normalized shear stress vs. time. The inset shows the relation between the final stress $\sigma _{fin}$, and the shear rate $\dot{\gamma }_{src}$, and the standard deviation of $\sigma _{fin}$ is included in the circle. (**b**) Normalized shear stress vs. strain.

The effect of shear rate imposed in SRC test, $\dot{\gamma }_{src}$, on the stress overshoot was investigated at five different $\dot{\gamma }_{src}$ with a same pre-rest condition ($t_{pr}$ = 60 min and $\dot{\gamma }_{pr}$ = 0 s^−1^). Figure 2a shows the relative stress to the final stress, $\sigma /\sigma _{fin}$ as a function of time. The overshoot appeared in all cases, but became more evident with an increase in $\dot{\gamma }_{src}$. The linear $\sigma _{fin}$ vs. $\dot{\gamma }_{src}$ in the inset denotes a similar tendency to the data of magma at the steady state^1,7,33^. Therefore, magma of which the steady-state characteristics are simple, has the potential to show much more complex rheology induced by aging in the non-steady state as in the case of this study. Further discussion on the steady state is described in Text S3 of the Supplementary Material. Figure 2b presents the relative stress vs. strain calculated from the time and shear rate. The relationships are similar, regardless of $\dot{\gamma }_{src}$, although the strain to reach the peak stress of the overshoot and the final stress increase with increasing shear rate.

### Microstructure

The experiments provided clear evidence of aging in the magma rheology as the stress overshoot in the start-up flow. The microstructural change related to the interaction between crystals during the pre-rest may cause the overshoot because whether it appears or not is dependent on the conditions, $t_{pr}$ and $\dot{\gamma }_{pr}$. To investigate the relation between the rheological and microstructural variations induced by aging, imaging with SEM and micro-CT technology was applied. Here we show the results of two representative samples; one is an unaged sample with $t_{pr}$ = 10 min and $\dot{\gamma }_{pr}$ = 0 s^−1^, and the other is a shear-aged sample with $t_{pr}$ = 60 min and $\dot{\gamma }_{pr}$ = 0.02 s^−1^, which showed the most prominent overshoot (Fig. 1b). The conditions and methods for the microstructural observations are described in Text S2 of the Supplementary Material.

#### SEM observations

**Figure 3:**
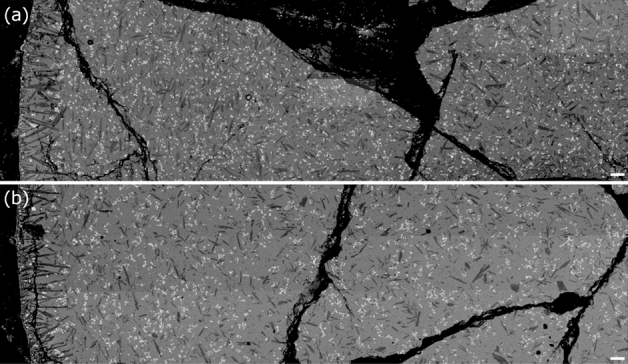
Back-scattered electron images of cross-sectional surfaces of samples cut off at a height of 13 mm from the bottom. The dark gray and elongated rectangles are plagioclase, the white crystals are Fe-Ti oxide, and the background is melt/glass. The scale bars in the lower right corners are 200 μm. Each figure is a composite image in which 27 images are superimposed. (**a**) Unaged sample with $t_{pr}$ = 10 min and $\dot{\gamma }_{pr}$ = 0 s^−1^. (**b**) Shear-aged sample with $t_{pr}$ = 60 min and $\dot{\gamma }_{pr}$ = 0.02 s^−1^.

SEM observations revealed that both of the unaged and shear-aged samples contain Fe-Ti oxide and plagioclase in the melt (Fig. 3). The crystal volume fractions $\phi$, estimated from 10 images using Fiji software^36^ are comparable; $\phi$ of Fe-Ti oxide is 0.07 ± 0.010 in the unaged sample and 0.07 ± 0.012 in the shear-aged sample, and $\phi$ of plagioclase is 0.09 ± 0.012 in the unaged sample and 0.09 ± 0.017 in the shear-aged sample. The crystal volume fraction does not change with aging; therefore, an increase in crystals is not the cause of the stress overshoot in the rheology. Compared with the critical crystal volume fraction $\phi _c$, at which a crystal network first forms determined by^13^, the total crystal volume fractions of the two samples (ca. 0.16) are within the range of $\phi _c$ for plagioclase, 0.08 $< \phi _c<$ 0.20, and close to $\phi _c$ for randomly-oriented cubes, 0.22. This is consistent with the results reported here, which show non-Newtonian rheology. With respect to the crystal alignments in Fig. 3, the crystals, Fe-Ti oxide in particular, do not exist alone but as aggregates, and the aggregates are larger and more noticeable in the shear-aged sample. Dense parts composed of the aggregated crystals are sparse in the shear-aged sample, where the estimated $\phi$ has a larger error than that of the unaged sample. It can be interpreted that contact of the aggregates may proceed by slightt shear during pre-rest to form clusters, and the clusters are broken after the onset of the SRC test by shear that causes the stress overshoot. A similar process of shear-induced memory effect on reversible clusters composed of several unbreakable aggregates of primary particles has recently been proposed in the field of soft glass materials^37^.

#### Micro-CT imaging

**Figure 4:**
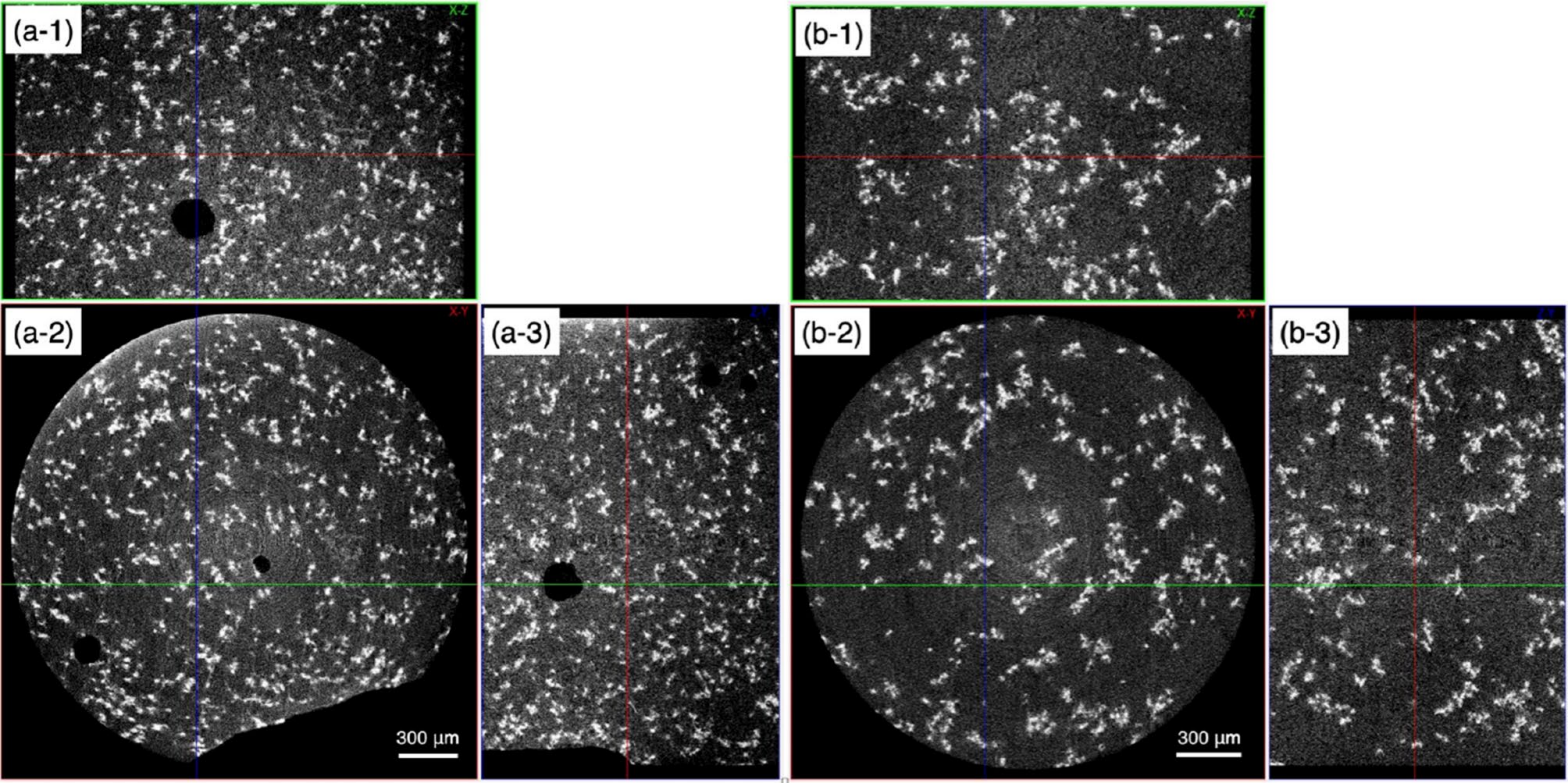
Micro-CT images of the same samples as those used for SEM observations. The white crystals are Fe-Ti oxide, the background is melt/glass and plagioclase. (**a**) Unaged sample with $t_{pr}$ = 10 min and $\dot{\gamma }_{pr}$ = 0 s^−1^. (**b**) Shear-aged sample with $t_{pr}$ = 60 min and $\dot{\gamma }_{pr}$ = 0.02 s^−1^. (**a-1**) and (**b-1**) are sagittal planes and (**a-3**) and (**b-3**) are coronal planes composed of 1531 images each. (**a-2**) and (**b-2**) are transverse planes.

To examine the 3D structure of the clusters grown by aging, analyses of micro-CT images were performed. Since the CT imaging cannot distinguish plagioclase from melt due to the subtle difference between the densities, here we focus on Fe-Ti oxide, of which 2D distribution strongly depended on aging (Fig. 3). In Fig. 4, large clusters of Fe-Ti oxide are present in all cross sections of the shear-aged sample, while small aggregates are more dispersed in the unaged sample. Image analyses were performed to obtain the quantitative characteristics of the clusters using Fiji software^36^ with MorpholibJ integrated library and plugin^38^. The detailed imaging conditions and the procedure for image processing are described in Text S2 of the Supplementary Material.

Table 1 shows the results of the image analyses. The number of objects detected is smaller while the volume is larger in the shear-aged sample than in the unaged sample. This indicates that pre-existent aggregates coalesce with time and become larger, thereby reducing their number during the pre-rest because the total volumes of Fe-Ti oxide in the two samples are equivalent. The results mean that aging affects not the volume of crystals but the crystal arrangement, which agrees well with the SEM observations. A reduction in sphericity would suggest that grown clusters have a greater variety of shape. Histograms of the original object volume and sphericity are shown in Fig. S2 of the Supplementary Material. With respect to the shape of equivalent ellipsoid, although the size increases with aging, the shape maintains a scaling relationship, which is evident in the normalized equivalent ellipsoid sizes. The aggregates are long in the shear-plane; therefore, it is easier for them to collide in the plane, so that residual shear-history before the pre-rest and weak shear during the pre-rest would promote the formation of clusters. On the other hand, the small aggregates dispersed in the unaged sample would be formed originally by the contact of crystals due to shear during crystallization and those may be scarcely broken by the shear imposed in this study. This could explain why the elevation of the major axis of the equivalent ellipsoids on the horizontal plane, or shear plane is close to zero. The effect of shear on crystal alignment has also been referred to in previous studies^24,39^.

Overall, this study concludes rheological changes are caused by forming crystal clusters by aging without changing characteristics of each crystal, so that this is different from the effects of crystal shape, size, and volume fraction^1,12,13,15,16^. We consider clustering is not caused by magnetic force between the oxides because the Curie temperature is much lower than our experimental temperature. Instead, shear-induced acceleration of synneusis, which is a hydrodynamic process that can drive the aggregation of preformed crystals^40–42^, is a possible mechanism of aging, although future works are required to explore the effect in detail.

**Table 1:** Summary of 3D structures of Fe-Ti oxide aggregates in the unaged sample and clusters in the shear-aged sample.

	Unaged sample	Shear-aged sample
Number of objects	4,183,887	903,772
Total volume of Fe-Ti oxide (μm^3^)	1.029$\cdot {10}^{8}$	1.012$\cdot {10}^{8}$
Volume fraction of Fe-Ti oxide	0.070	0.069
Object volume (μm^3^)	4615	11543
Object sphericity	0.353	0.290
Equivalent ellipsoid size (μm)	17.70, 10.89, 7.60	23.18, 14.79, 10.61
Normalized equivalent ellipsoid size (μm)	1, 0.62, 0.43	1, 0.64, 0.46
Elevation of the major axis on the horizontal plane ($^{\circ }$)	− 0.66	0.77

The five characteristics in the lower panel are median values from the histograms.

### Implication for magma flow inside volcanic conduit

This study shows that the peak stress of overshoot in the start-up flow increases if the magma has sufficient time at rest, and low shear rates promote the process. The temporal variation in magma rheology caused by aging would affect the dynamics of magma and lava associated with an eruption. An example is the formation of a high-viscosity plug in a shallow conduit, which would significantly affect the dynamics of Strombolian eruptions^43–45^. Aging would prompt plug formation as follows; (1) magma stops its ascent after degassing. (2) At the shallow conduit depth, the arrangement of crystals changes with time and form clusters by aging, which leads to yield stress. (3) Due to the yield stress, the stagnant magma separates from the main convection flow in the conduit. (4) Cooling and crystallization are facilitated in the stagnant magma. The process becomes dominant in the presence of Fe-Ti oxide since the liquidus increases under the oxidized state^46^. As a result, a high-viscosity plug is formed, increasing the explosivity of eruption. In this way, aging would play an important role during the initial stage of the plug formation. Although Fe-oxides, of which clusters were formed in our shear-aged sample, are less likely to observe at natural systems, this scenario is possible at shallow parts of volcanic conduit because there would be an oxidized state with oxidized recycled materials^46,47^ and magnetite nanolite^48^. Since magnetite nanolites could drastically increase the viscosity^49^, it makes a plug more viscous, and the structure becomes even stronger by aging, causing more explosive eruptions. We expect aging in other crystals, too. Especially, since clusters of plagioclase have been observed in natural products^42^ and experimental samples^25^, aging may affect the formation. That would be a next key question to address in future work.

## Conclusions

Rheological measurements and microstructural observations were performed on aphyric basaltic andesite lava produced by the 1986 Izu-Oshima eruption. The rheological measurements indicated the overshoot of shear stress in the start-up flow by aging with sufficient pre-rest time, which became predominant in the case of imposing weak shear during the pre-rest. The microstructural observations revealed that original aggregates of crystals, which grew during crystallization, coalesce during the pre-rest to form clusters, and the clusters are broken by shear in the start-up flow, which induces the stress overshoot. The temporal variation in magma rheology caused by aging would prompt the formation of high-viscosity plug of magma flow inside a volcanic conduit and would control the onset/stop of magma and lava flows.

## Supplementary Information

Supplementary Information.

## Data Availability

The datasets used and analyzed during the current study are available from the corresponding author, AK on reasonable request.
