# Supplementary material for: Aging in magma rheology
Source: Sci Rep. 2022 Jun 15;12:10015. doi: 10.1038/s41598-022-14327-2 (PMC9200731; doi:10.1038/s41598-022-14327-2)
Supplement: Supplementary file 1 — Supplementary Information. [file 41598_2022_14327_MOESM1_ESM.docx]

Supplementary Material for: Aging in Magma Rheology

Aika K Kurokawaa,∗, Takahiro Miwaa, and Hidemi Ishibashib

*aNational Research Institute for Earth Science and Disaster Resilience, 3-1 Tennodai, Tsukuba, 3050006, Ibaraki, Japan*

*bDepartment of Geosciences, Faculty of Science, Shizuoka University, 836 Ohya, Suruga-ward, 4228017, Shizuoka, Japan*

This supporting material provides the following contents.

**Text S1**: Analytical method for rheological data
**Text S2**: Conditions and procedures for SEM observation and micro-CT imaging
**Text S3**: Rheological behavior at steady state
**Table S1**: Elemental compositions of LB natural product and experimental samples
**Figure S1**: Stress change in the process of crystallization and stabilization
**Figure S2**: Histograms of volume and sphericity used for image analyses

- **Text S1**

Shear stress, $\sigma$ is given by:

$\sigma=\frac{T}{2\pi R_{r}^{2}L}$ *,*

where $T$ is the torque measured by the viscometer, $\omega$ is the angular velocity, $R_{r}$ is the radius of the rod, 2.5 mm, and $L$ is the sample height, 15 mm. To convert the angular velocity into the shear rate at the wall of rod, $\dot{\gamma}$, we use the following equation proposed by Margules (1881):

$\dot{\gamma}=\frac{{2\omega R}_{c}^{2}}{R_{c}^{2}-R_{r}^{2}}$ ,

where $R_{c}$ is the radius of the container, 9.5 mm. Although the alumina container reacts with the lava sample within the width of ~ 0.6 mm as in Figure 3 in the main manuscript, it causes a negligible error of ~ 1 % in the shear rate. The measurement precision was calibrated using a viscosity standard (oil JS14000) at 20ºC and LB melt at 1200ºC. The series of experiments was repeated more than once, and we confirmed that the aging dynamics are reproducible for the given protocol.

- **Text S2**Quenched samples used in the rheological measurements were polished after cutting and carbon coated for electron microscopy. Backscattered electron images shown in Figure 3 of the main manuscript were collected at a magnification of 90 with an acceleration voltage of 15 kV and a beam current of 60 nA using JSM-IT500 scanning electron microscope (JEOL Ltd.) at National Research Institute for Earth Science and Disaster Resilience.

3D imaging by X-ray CT was performed using SkyScan 1272 (Bruker) with a 90 kV-110 μA energy source and 360º rotation in steps of 0.2º. The spatial resolution was 0.9 μm/voxel. In the case of scanning the shear-aged sample, a Al0.5 mm + Cu0.038 mm filter was used. 3D image analyses were performed using Fiji open-source software (Schindelin et al., 2012) with MorpholibJ integrated library and plugin (Legland et al., 2016). First, a cylindrical sample was cut into a quarter cylinder of which the size is 1226×1226×120 voxels in the images, because it was difficult to analyze the original images due to the lack of space to download such a large volume of data. Figure 4 of the main manuscript highlights that this approach is reasonable, as there is no heterogeneity within the sample. Second, Gaussian and median filters were applied for noise reduction, and the threshold of brightness was determined to detect Fe-Ti oxide. After the grayscale images were converted into binary images, objects were then detected using an open-source distance transform watershed 3D method (Legland et al., 2016), which involves calculating the distance transform of the binary image, inverting it and then applying watershed to it using the original image as a mask. The object shapes were finally obtained by fitting equivalent ellipsoids to the volumes. Each ellipsoid is triaxial and the three axes pass through the　spatial center of the object.

- **Text S3**

Here we discuss the result at the steady state shown in the inset of Figure 2(a) in the manuscript by focusing on the relative viscosity, $\eta_{r}=\eta/{\mu_{0}}$, which is the apparent viscosity $\eta$ normalized by the melt viscosity $\mu_{0}$. The melt viscosity was calculated as 1798.5 Pa・s using the equations reported in Giordano et al. (2008) with the chemical composition of melt in the shear-aged sample shown in Table S1. Although the relative viscosity decreases with increasing the shear rate showing shear-thinning, the value is within the range of 1.2-1.3 at the shear rates imposed in this study (0.68-2.48 s^-1^). Considering the crystal volume fraction of ~ 0.16, the relative viscosity is consistent with common models of particle suspension rheology (Mader et al., 2013). Especially, it is equivalent to Einstein-Roscoe equation for spherical particles (Roscoe, 1952) and experimental results of magma contained olivine and plagioclase measured by Ishibashi and Sato (2007). This indicates that elongate clusters formed in the pre-rest time by aging are broken through the stress overshoot, and the sample at the steady state can be regarded as suspension of round particles. The microstructural change would occur during each SRC test, leading to the correspondence of final stress, $\sigma_{fin}$ regardless of aging conditions.

- **Table S1**
  Element analyses of LB natural product and experimental sample were performed by using a X-ray fluorescence spectrometer (ZSX PrimusⅡ, Rigaku Co., Ltd.) at Earthquake Research Institute in the University of Tokyo. More detailed information for the analytical method is described in Hokanishi et al., (2015). On the other hand, the chemical data of melt in the shear aged sample was obtained by using SEM coupled with energy dispersive spectroscopy (Ultim Max 65, Oxford Instruments) at National Research Institute for Earth Science and Disaster Resilience. Each analysis was performed with an acceleration voltage of 15 kV and a beam current of 0.6 nA for 40 s to an area of which the size is 5 μm×5 μm in the sample. The data shown in Table S1 are average values of 17 areas.

|  | Natural product | Experimental sample | Melt in shear-aged sample |
| --- | --- | --- | --- |
| Oxide | wt.% | wt.% | wt.% |
| SiO2 | 54.97 | 55.00 | 58.97 |
| TiO2 | 1.21 | 1.21 | 1.37 |
| Al2O3 | 14.26 | 15.60 | 15.08 |
| FeO | 13.97 | 13.04 | 9.10 |
| MnO | 0.23 | 0.22 | 0.25 |
| MgO | 3.76 | 3.68 | 3.95 |
| CaO | 8.67 | 8.35 | 8.24 |
| Na2O | 2.26 | 2.22 | 2.36 |
| K2O | 0.54 | 0.56 | 0.59 |
| P2O5 | 0.13 | 0.12 | 0.08 |
| Total | 100.00 | 100.00 | 100.00 |

- **Figure S1**Stress change at 1.13 s^-1^ from the middle of (2) to the end of (3) in the experimental protocol described in the main manuscript. In the figure, t = 0 corresponds to when the temperature was lowered from 1300ºC to the experimental temperature of 1180ºC. As the two results approximately match, the process was reproducible. **
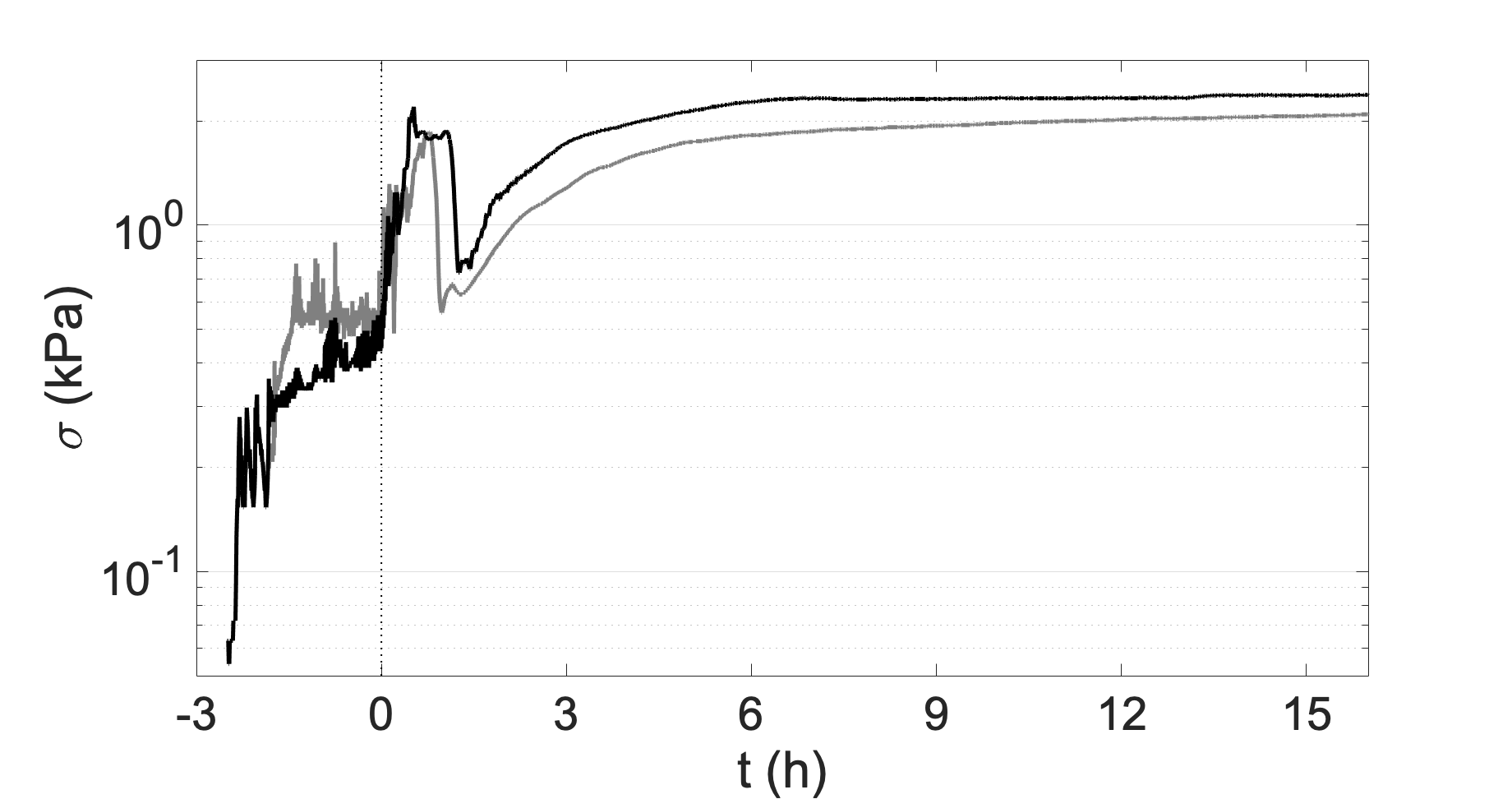
**
- **Figure S2**Normalized histograms of (a) cluster volume and (b) sphericity for the unaged sample in blue and the shear-aged sample in red. The volume of the shear-aged sample becomes larger than that of the unaged sample, whereas the sphericity decreases, which indicates cluster growth with aging.

**References**

Giordano, D., Dingwell, D.B., Non-Arrhenian multicomponent melt viscosity: a model, Earth and Planetary Science Letters 208, 337-349 (2003). https://doi.org/10.1016/S0012-821X(03)00042-6

Hokanishi, N., Yasuda, A., Nakada, S., Major and trace element analysis of silicate rocks using fused glass beads with an X-ray fluorescence spectrometer. (in Japanese with English abstract), Bull. Earthq. Res. Inst. Univ. Tokyo 90, 1-14 (2015).

Ishibashi, H., Sato, H., Viscosity measurements of subliquidus magmas: Alkali olivine basalt from the Higashi-Matsuura district, Southwest Japan, Journal of Volcanology and Geothermal Research 160(3-4), 223-238 (2007). https://doi.org/10.1016/j.jvolgeores.2006.10.001

Legland, D., Arganda-Carreras, I., Andrey, P., MorphoLibJ: Integrated library and plugins for mathematical morphology with ImageJ. Bioinformatics 32(22), 3532–3534 (2016). https://doi.org/10.1093/bioinformatics/btw413

Mader, H.M., Llewellin, E.W., Mueller, S.P., The rheology of two-phase magmas: A review and analysis. Journal of Volcanology and Geothermal Research 257, 135-158 (2013). https://doi.org/10.1016/j.jvolgeores.2013.02.014

Margules, M., On the determination of the coefficients of friction of friction and of gliding in the plane motions of a fluid, Wien. Akad. Sitzber., [Ser. 2] 83, 588-602 (1881) (in German).

Roscoe, R., The viscosity of suspensions of rigid spheres. British Journal of Applied Physics 3, 267-269 (1952)

Schindelin, J., Arganda-Carreras, I., Frise, E., Al., E., Fiji: an open-source platform for biological-image analysis. Nature Methods 9, 676–682 (2012). https://doi.org/10.1038/nmeth.2019
